# Cut-and-Paste of DNA Using an Artificial Restriction DNA Cutter

**DOI:** 10.3390/ijms14023343

**Published:** 2013-02-05

**Authors:** Makoto Komiyama

**Affiliations:** Life Science Center of Tsukuba Advanced Research Alliance, University of Tsukuba, Tsukuba, Ibaraki 305-8577, Japan; E-Mail: komiyama@tara.tsukuba.ac.jp

**Keywords:** site-selective scission, DNA cutter, Ce(IV)/EDTA, PNA, human genome, homologous recombination, restriction enzyme-free manipulation

## Abstract

DNA manipulations using a completely chemistry-based DNA cutter (ARCUT) have been reviewed. This cutter, recently developed by the authors, is composed of Ce(IV)/EDTA complex and two strands of pseudo-complementary peptide nucleic acid. The site-selective scission proceeds *via* hydrolysis of targeted phosphodiester linkages, so that the resultant scission fragments can be easily ligated with other fragments by using DNA ligase. Importantly, scission-site and site-specificity of the cutter are freely tuned in terms of the Watson–Crick rule. Thus, when one should like to manipulate DNA according to the need, he or she does not have to think about (1) whether appropriate “restriction enzyme sites” exist near the manipulation site and (2) whether the site-specificity of the restriction enzymes, if any, are sufficient to cut only the aimed position without chopping the DNA at non-targeted sites. Even the human genome can be manipulated, since ARCUT can cut the genome at only one predetermined site. Furthermore, the cutter is useful to promote homologous recombination in human cells, converting a site to desired sequence. The ARCUT-based DNA manipulation should be promising for versatile applications.

## 1. Introduction

Current biotechnology is primarily based on cut-and-paste of plasmid DNA in which restriction enzymes and DNA ligase are used. However, most of naturally occurring restriction enzymes recognize only 4–8 base-pair sequences so that their scission sites statistically appear at every 4^4^ (=256), 4^6^ (=4096), and 4^8^ (=65,536) base-pair sequences, respectively. In order to cut (and manipulate) huge genomic DNA precisely, this site-specificity is far from satisfactory. Thus, site-selective and position-free DNA cutters have been strongly desired to develop relevant fields furthermore [1–6]. Despite many attempts through chemical and biological approaches, however, DNA cutters, which have high site-specificity and can be used to manipulate huge DNA, have not been available for a long time. Furthermore, even when much smaller DNA of conventional size (e.g., 5 kbp) is the target of manipulation, what often happens is that appropriate restriction enzyme to cut the DNA at desired position cannot be found and thus another pathway, in which the second-choice (or the third-choice) restriction enzymes are used, must be employed. Accordingly, freedom in the choice of selective scission site is also important for DNA cutters to be widely applicable to versatile purposes in the relevant fields.

Recently, our group prepared an artificial restriction DNA cutter (ARCUT), which is completely chemistry-based and cuts double-stranded DNA at the desired site with aimed site-specificity [1–7]. There, Ce(IV)/EDTA complex is used as molecular scissors for hydrolytic scission of DNA, whereas the target sequence is recognized by pseudo-complementary peptide nucleic acid (pcPNA) through Watson-Crick rule. One of the most important characteristics of this cutter is that it cuts DNA via hydrolysis of targeted phosphodiester linkages exactly as naturally occurring nucleases do. Thus, the DNA fragments obtained by the ARCUT-based site-selective scission can be connected with other DNA fragments by using the DNA ligase, exactly as is done in conventional molecular biology. This ARCUT-based cut-and-paste technology is the main theme of this review.

It is also noteworthy here that, in addition to these completely chemistry-based DNA cutters, several kinds of protein-based site-selective DNA cutters have recently been developed [8,9]. There, DNA-cutting module of naturally occurring nucleases (or the catalytic center of restriction enzymes) is conjugated with other proteins, which selectively bind to the target sequence in DNA (e.g., zinc finger proteins and transcription activator-like effector proteins). The developments of these protein-based cutters are remarkable in this decade, and they are highly promising for future applications, especially for gene targeting in cells. However, detailed description on these protein-based cutters is beyond the scope of this review, since this manuscript will only concentrate to chemistry-based DNA cutters. The readers who are interested in the latter type of DNA cutters are advised to refer to the corresponding references. Chemical tools, which cut DNA site-selectively through oxidative cleavage, are not described here either because of space limitation and the reader should refer to a recent review [1].

## 2. Molecular Design of Completely Chemistry-Based Artificial Restriction DNA Cutter (ARCUT)

**Figure 1:**
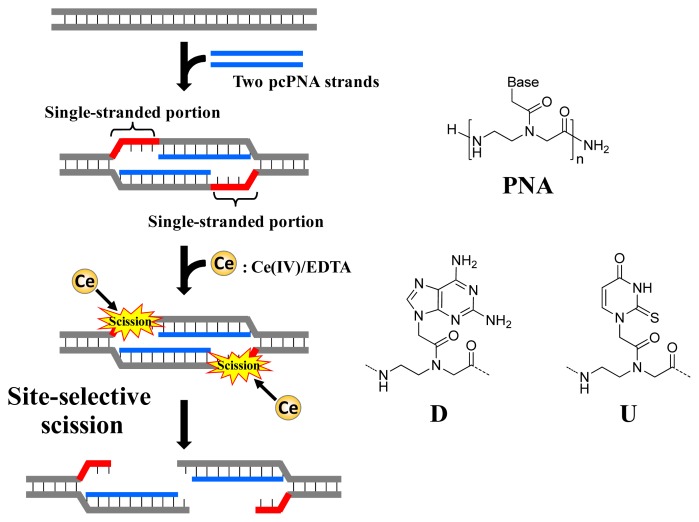
Site-selective scission of double-stranded DNA by an artificial restriction DNA cutter (ARCUT). Two pcPNA strands (blue lines) invade DNA substrate to form single-stranded portions (red parts) in both DNA strands. The phosphodiester linkages in the single-stranded portions are hydrolyzed by Ce(IV)/EDTA. The structure of pcPNA is presented in the right.

As depicted in Figure 1, ARCUT is composed of Ce(IV)/EDTA complex and pseudo-complementary peptide nucleic acid (pcPNA). The Ce(IV)/EDTA complex as molecular scissors has a remarkable substrate-specificity in that it promptly hydrolyzes the phosphodiester linkages in single-stranded DNA but hardly hydrolyzes the linkages in double-stranded DNA [10]. On the other hand, pcPNA is a DNA analog in which the phosphodiester linkages are replaced by amide linkages (see the right-hand part of Figure 1) [11–13]. In order to promote the invasion to double-stranded DNA, modified nucleobases 2,6-diaminopurine (D) and 2-thiouracil (U) are used in place of conventional A and T bases. The mechanism of site-selective hydrolysis of double-stranded DNA by ARCUT is schematically presented in Figure 1. First, two pcPNA strands (the blue lines) bind to the complementary sequence in the DNA according to Watson–Crick rule, and form so-called double-duplex invasion complex. It should be noted that the binding sites of these two pcPNAs are designed to be laterally shifted to one another by several nucleobases. Thus, in the invasion complex, unpaired single-stranded portions (the red parts) are formed at the predetermined sites in both strands of the double-stranded DNA. These single-stranded portions are selectively hydrolyzed by the Ce(IV)/EDTA, since this complex hydrolyzes only single-stranded DNA.

In order to accelerate the DNA scission furthermore, a phosphoserine residue should be attached to the terminus of each of the two pcPNA strands. In the invasion complex (in Figure 1), these monophosphate groups are located near the targeted scission site (the single-stranded portions in red) and strongly attract Ce(IV)/EDTA. As the result, the local concentration of the molecular scissors at the target site is increased and the site-selective DNA scission is remarkably promoted. Throughout this review, this type of ARCUT (Ce(IV)/EDTA + two strands of pcPNA bearing a monophosphate) will be used.

## 3. Cutting DNA: Site-Selective Scission of Double-Stranded DNA by ARCUT

**Figure 2:**
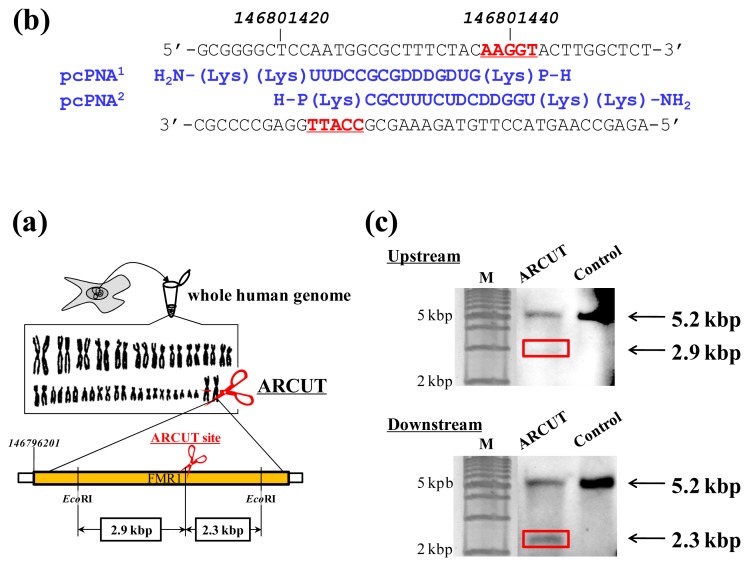
Site-selective scission of the human genome by ARCUT. (**a**) The scheme of scission of one site in FMR1 gene in the X chromosome. After the ARCUT reaction, the product was digested with *Eco*RI and analyzed by Southern blotting. The pcPNA strands used and the results of Southern blotting are shown in (**b**) and (**c**). The bands at 2.9 kbp (the upper gel) and 2.3 kbp (the lower gel) correspond to the DNA fragments formed by the dual scission by the ARCUT and *Eco*RI. On the other hand, the band at 5.2 kbp comes from the dual scission by *Eco*RI alone (without the scission by the ARCUT).

ARCUT can cut double-stranded DNA of various sizes (from plasmid to human genome) at any chosen target site. The DNA scission is completely hydrolytic. In Figure 2, for example, one site in the human genome (3 × 10^9^ bp) was selectively hydrolyzed [14]. As the targeted scission site, one site in FMR1 (Fragile X Mental Retardation 1) gene in the X chromosome was chosen (this choice is arbitrary). This 38-kbp gene is highly conserved and involves CGG trinucleotide repeats in its 5′ untranslated region. If the number of this repeat exceeds 200 and abnormal hypermethylation occurs, the transcription of protein (FMRP) from this gene is silenced and fragile X syndrome is induced. For the site-selective scission by ARCUT, the whole human genome was isolated from cultured human cells, and first incubated with 1:1 combination of pcPNAs. One of the pcPNA strands is complementary to A146801423-C146801437 in the upper strand of FMR1 gene, and another pcPNA is complementary to G146801428-A146801442 in the lower strand (Figure 2a). Accordingly, in the invasion complex, A146801438-T146801442 in the upper strand of the gene and T146801423-C146801427 in the lower strand became single-stranded. After the hydrolysis by Ce(IV)/EDTA, the ARCUT scission product was further treated with a restriction enzyme *Eco*RI and analyzed by Southern blotting to confirm the targeted site-selective scission. If the scission by this ARCUT, as designed, selectively occurred at the target site in the *FMR1* gene, 2.9 kbp fragment (upstream of the scission site) and 2.3 kbp fragment (downstream of the scission site) should be formed (Figure 2b). In fact, these two fragments were clearly detected by Southern blotting using the corresponding probes (Figure 2c). As expected, The scission efficiency increased in proportion to the Ce(IV)/EDTA concentration. Importantly, other analogous sequences in the human genome were never cut by this ARCUT (see below). This absence of undesired off-target scission should be very important, especially when these cutters are used *in vivo* (e.g., for therapy).

## 4. Mismatch-Recognition in Site-Selective Scission by ARCUT

**Figure 3:**
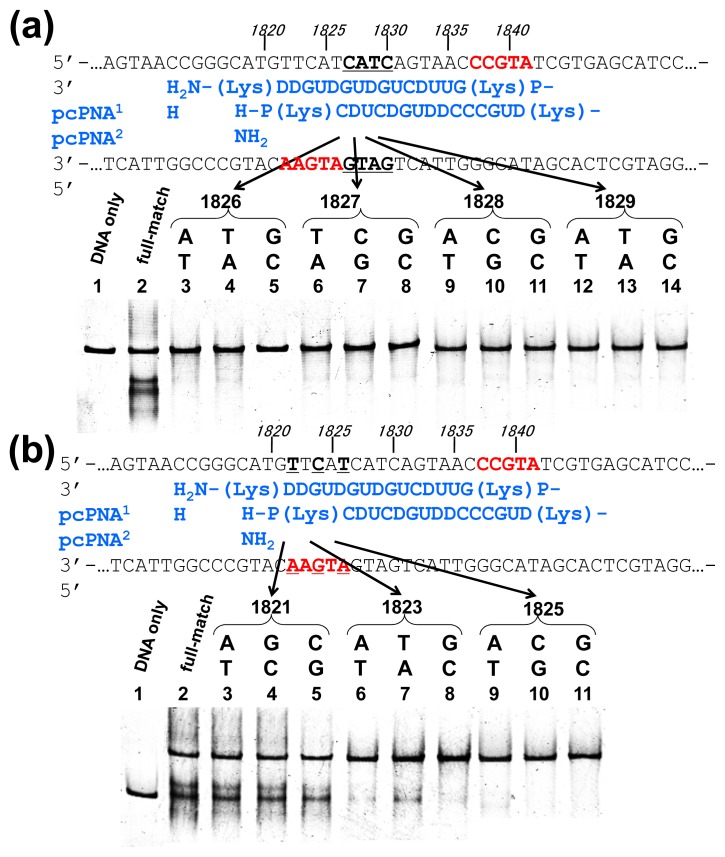
Mismatch recognition of ARCUT for site-selective scission of double-stranded DNA. (**a**) Mismatches introduced in the central double-invasion region and (**b**) mismatches introduced in the flanking single-invasion region. In both (**a**) and (**b**), lane 2 is for fully matched DNA, and in other lanes, one base-pair (underlined) was changed to another base-pair as indicated. [DNA] = 20 nM, [each of pcPNAs] = 100 nM, [Ce(IV)/EDTA] = 50 μM, [NaCl] = 100 mM, and [HEPES (pH 7.0)] = 5 mM at 50 °C for 14 h.

Off-target scission, if any, should often induce fatal results in clinical applications and others. Thus, the mismatch-discrimination ability of ARCUT was comprehensively investigated in detail in Figure 3 [15]. A typical ARCUT, in which 15-mer pcPNAs are laterally shifted by 5 nucleobases, was employed. One base-pair in substrate DNA was systematically changed to another pair, and the effect of introduced mismatch(es) on the scission efficiency was analyzed. By replacing a CG pair at 1826 with AT pair, for example, the ARCUT scission completely disappeared. Similar results were obtained by introducing TA or GC pair to this position in place of CG pair. These analyses were made throughout the invasion region.

These systematic studies gave the following conclusion. The 10 base-pairs in the middle region in the invasion complex, where two pcPNAs simultaneously bind to both of the two DNA strands, are recognized almost perfectly (Figure 3a). In other words, alteration of only one base-pair in this region to another pair completely diminishes the scission by the ARCUT. Note that alteration of one base-pair in the double-stranded DNA intrinsically induces two mismatches to the whole system (one mismatch is between the upper strand of the DNA and one pcPNA strand, and another mismatch is between the lower strand and another pcPNA). On the other hand, the recognition in the flanking terminal region, where only one pcPNA binds to the DNA, is less strict (Figure 3b). Even when one-base pair in this region (especially at the end of the invasion site) is changed to another, the scission occurs to some extent. In total, 16 base-pairs in the target sequence (terminal 3 + internal 10 + terminal 3) are recognized by this ARCUT in an almost completely on-off way. Accordingly, from statistical viewpoints, the scission site of this ARCUT should appear at every 4^16^ (=4 × 10^9^) bp, which is larger than the size of human genome (3 × 10^9^ bp). Of course, when necessary, the site-specificity can be further increased by using still longer pcPNAs. High potential of ARCUT for gene-targeting in human cells is strongly evidenced.

**Figure 4:**
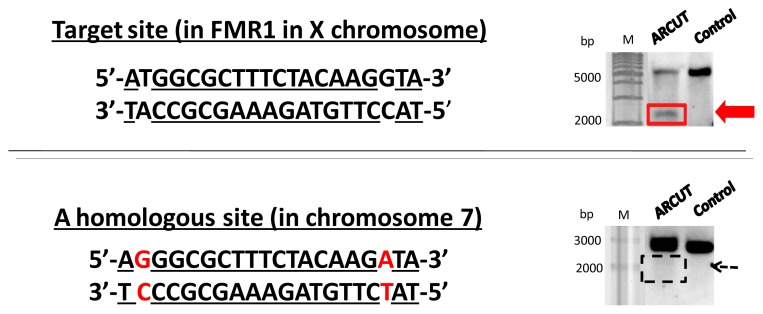
Strict recognition of ARCUT in the site-selective scission of human genome. The ARCUT targeting FMR1 in X chromosome never cuts the analogous site in chromosome 7. Note that the underlined parts are the same in these two sites.

Remarkably, high mismatch-recognizing activity of ARCUT was directly confirmed by the result in site-selective scission of human genome [9]. In the human genome, there are many analogous sites, which are different from each other by only one or two base-pairs in the sequence (e.g., in 20-bp length). However, it was confirmed that ARCUT strictly distinguishes the target site from highly analogous sequences and cuts only the former. For example, the ARCUT which is designed to target a site in FMR1 cuts this site satisfactorily (Figure 2). There are so many analogous sites in the human genome, but one site in chromosome 7 is exceptionally similar (Figure 4). Thus, the 15-bp sequence underlined is exactly the same as the target one in the *FMR1* gene. Other three flanking base-pairs are also the same. With the ARCUT designed for the scission of FMR1, however, this analogous site in chromosome 7 was strictly distinguished from the target site and was never hydrolyzed.

## 5. Pasting DNA Fragments: Ligation of ARCUT Scission Fragment with Another Fragment to Provide a Recombinant DNA

As described above, the DNA scission by ARCUT proceeds via hydrolysis of targeted phosphodiester linkages and thus the resultant DNA fragments can be directly connected with other DNA fragments by using DNA ligase. This availability to cut-and-paste method is a great advantage of ARCUT as a tool for biotechnology and molecular biology. In this section, the methods for manipulation of plasmid are mainly described because of its simplicity. However, ARCUT-based cut-and-paste technology is never restricted to plasmid manipulation and is available for versatile purposes (even human genome can be manipulated).

It should be noted here that there is an important but very simple trick to achieve the ligation of scission fragments efficiently. It is just to add appropriate oligonucleotide (“jointing oligonucleotide”) as the additive to the ligation mixture. In the DNA scission by ARCUT, the phosphodiester linkages in the single-stranded portions, formed at the target site by the invasion of two pcPNA strands, are hydrolyzed (see Figure 1). These portions involve several phosphodiester linkages, which are cut by the Ce(IV)/EDTA at similar rates. Thus, the scission product of ARCUT is a mixture of several fragments which are different from each other in the terminus structure (the number of protruding nucleotides is different). In order to prepare predetermined recombinant DNA, only one predetermined fragment must be picked up from the mixture and combined with the foreign fragment. As evidenced below, this task is easily achievable, simply by adding “jointing oligonucleotide” to the ligation mixture, and desired recombinant DNA is selectively obtained [16].

**Figure 5:**
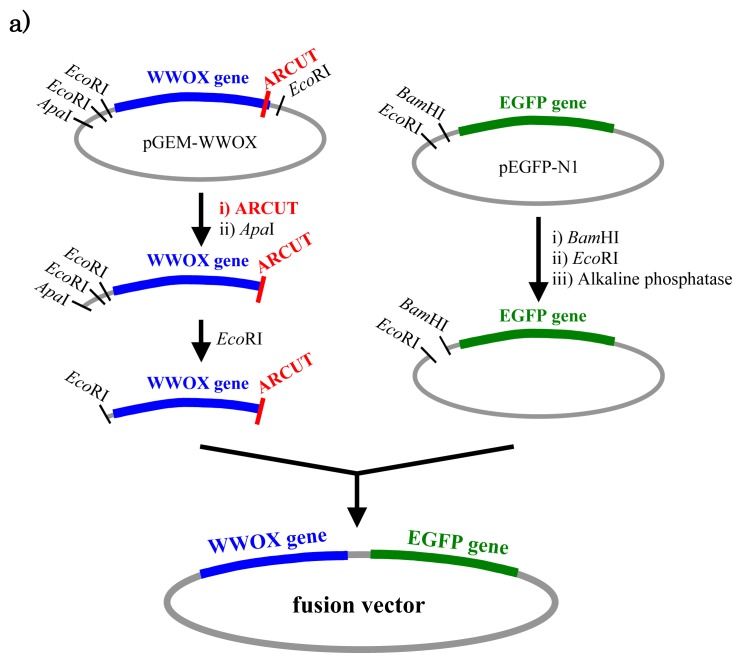

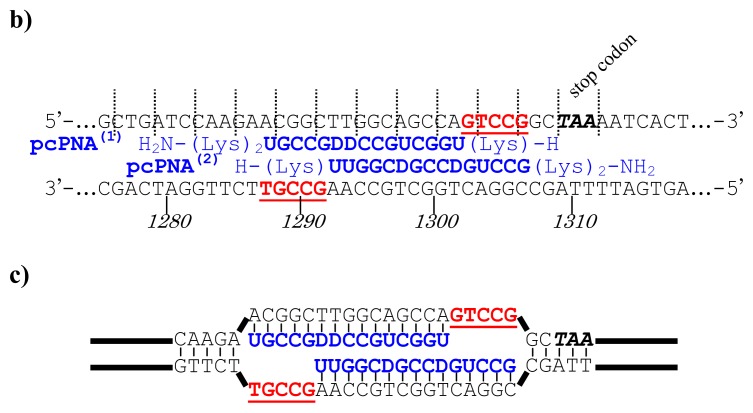
Outline of the construction of fusion protein (**a**). In (**b**), the sequences of substrate DNA at the ARCUT scission site and the pcPNA additives (in blue bold type) are shown. The single-stranded portions in both strands of the DNA (underlined red parts in (**b**) and (**c**)) are the hot spots and selectively hydrolyzed.

**Figure 6:**
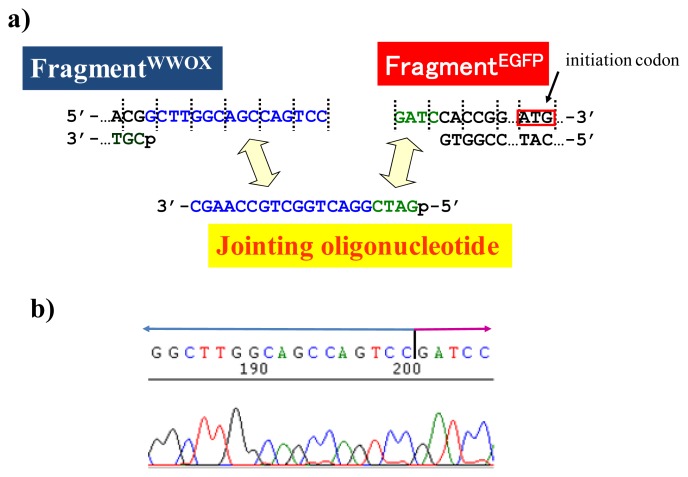
Ligation of the Fragment^WWOX^ and the Fragment^EGFP^. In order to ligate these two fragments, short jointing oligonucleotide is used (**a**). The reading-frames of *WWOX* gene and *EGFP* gene coincide each other as shown by the dotted lines. In (**b**), the sequence analysis of the cloned fusion vector at the conjunction is shown. The square represents the initiation codon of *EGFP* gene. The ligation conditions: 16 °C for 4 h.

In Figure 5, a cancer-related protein WW-domain-containing oxidoreductase (WWOX) was fused with enhanced green fluorescent protein (EGFP) by using ARCUT-based cut-and-paste method. There are three key points in this manipulation. The first one is to cut the *WWOX* gene just before its stop codon (TAA at 1310-1312). However, no “restriction enzyme sites” were available near this stop codon as the researchers often experience in DNA manipulation. This point was easily solved by designing ARCUT for the targeted site-selective scission. The second key point is how to ligate ARCUT fragment and EGFP fragment that have non-complementary termini each other. The last one is how to adjust reading-frame of the WWOX gene fragment with that of the fragment from EGFP. This adjustment is essential to express biologically active and fluorescently labeled fusion protein. The latter two requirements were successfully fulfilled by using appropriate oligonucleotide additive (jointing oligonucleotide in Figure 6) in the ligation step.

As depicted in Figure 5a, the gene of WWOX protein was first cut by the corresponding ARCUT at one site just before the stop codon (C1306-G1307 in the upper strand and C1290-C1291 in its lower strand). The sequences of the DNA and the pcPNA, as well as the structure of invasion complex, are presented in Figure 5 b,c. The dotted lines in Figure 5b show the reading-frames of the WWOX. On the other hand, the fragment of EGFP was prepared by cutting the gene with a restriction enzyme *Bam*HI just before the initiation codon. The downstream end of the WWOX fragment involves protruding upper strand (16-base sequence), whereas the upstream end of the EGFP fragment is *Bam*HI-scission terminus. Apparently, the terminal structures of these fragments are never complementary each other and inappropriate for direct enzymatic ligation with the use of DNA ligase. Accordingly, a jointing oligonucleotide (the Oligo^joint^ in Figure 6b: 20-mer), which forms completely cohesive end-structures with these fragments (the blue part and the green part), was added to the solution. In the presence of this Oligo^joint^, the enzymatic ligation promptly proceeded and the fusion gene (Fragment^WWOX^/Oligo^joint^/Fragment^EGFP^) was efficiently obtained. The ligation conditions employed were the same as used for conventional ligation. The sequencing results are presented in Figure 6b. Exactly as designed, *WWOX* gene and *EGFP* gene were directly connected with the reading-frames matched (see the vertical dotted lines). No mutation, addition, and deletion occurred in the DNA during the manipulation. The target fusion gene (and thus the fusion protein of designated primary sequence) has been successfully obtained. Other recombinant DNAs, which should be formed if the fragment other than Fragment^WWOX^ was ligated with Fragment^EGFP^, were not detected. Of the several scission fragments in the ARCUT reaction mixture, only the required fragment was incorporated to the recombinant DNA. As an alternative pathway, the fragments that have the same lower strand as this fragment but shorter upper strand could be ligated with the EGFP fragment, and the unpaired parts could be enzymatically repaired in *E. coli* to give the recombinant DNA of the same sequence. In any case, other scission fragments providing undesired recombinant DNA were left in the solution without being ligated with the EGFP fragment. When the fusion gene was transfected into mammalian cells (Cos-7), the protein was successfully expressed and clearly emitted green fluorescence. The DNA was never subjected to any substantial damage during the present ARCUT-based cut-and-past manipulation. The present fusion protein was primarily located around nuclei. It has been reported that *WWOX* localizes in Golgi apparatus under the conditions, whereas EGFP is mainly located in nuclei. In this case, the intracellular localization of the fusion protein was primarily governed by the EGFP portion.

**Figure 7:**
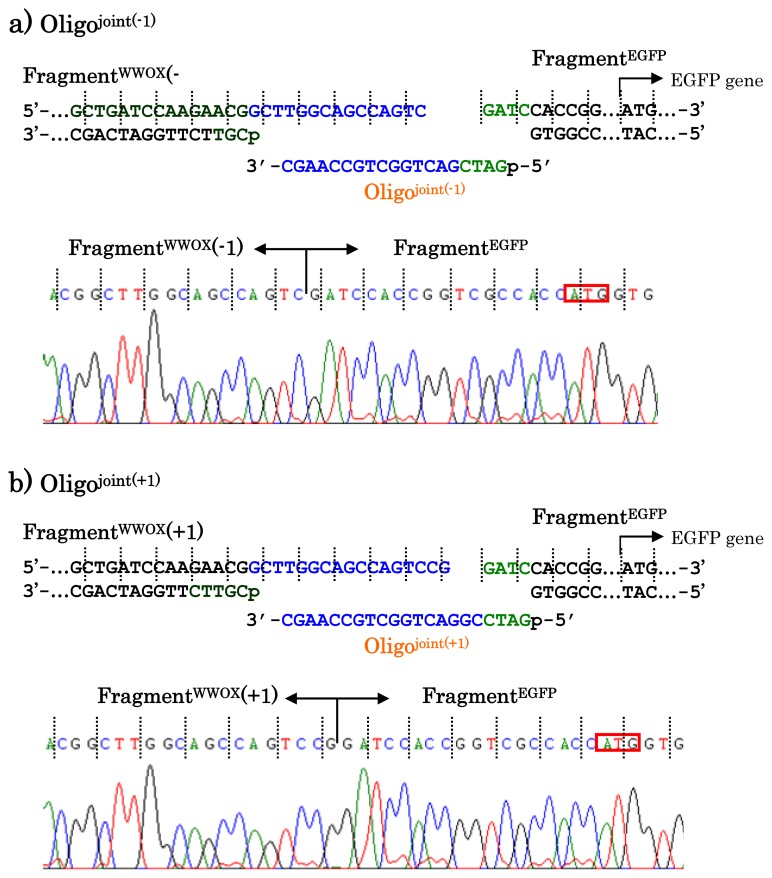
Enzymatic ligation of the ARCUT scission fragments with the Fragment^EGFP^ in the presence of Oligo^joint(−1)^ (**a**) and Oligo^joint(+1)^ (**b**).

When other jointing oligonucleotides were used in place of the Oligo^joint^, the corresponding scission fragment was selectively incorporated to the recombinant DNA (Figure 7). This is quite important from the viewpoint of practical applications. For example, a jointing oligonucleotide Oligo^joint(−1)^ in Figure 7a is one-base shorter than the Oligo^joint^, and thus its 3′-portion is complementary with another scission product Fragment^WWOX(−1)^ which is one-base shorter than the Fragment^WWOX^. As clearly shown by the sequencing experiments, the Fragment^WWOX(−1)^ was selectively picked up from the ARCUT reaction mixture and incorporated into the recombinant DNA (Fragment^WWOX(−1)^/Oligo^joint(−1)^/Fragment^EGFP^). In Figure 7b, Oligo^joint(+1)^ is one-base longer than the Oligo^joint^. Thus, the Fragment^WWOX(+1)^, which is one-base longer than the Fragment^WWOX^, was chosen, and Fragment^WWOX(+1)^/Oligo^joint(+1)^/Fragment^EGFP^ recombinant DNA was selectively formed. It is concluded that any desired recombinant DNA can be selectively formed by using appropriate jointing oligonucleotide. Although these two recombinant DNAs have wrong reading-frames and never produce fluorescent proteins (thus are meaningless for the formation of the fusion protein here), the potential of ARCUT for versatile applications has been completely confirmed.

When necessary, DNA fragments that can be directly ligated with ARCUT product (without the use of “jointing oligonucleotide”) can be prepared by use of “light-assisted cohesive-ending PCR” (LACE-PCR) [17]. In order to form a terminus in which one strand is protruding the other by 10–20 nucleotides like ARCUT product, photo-caged nucleotides are incorporated to the primers used for the PCR. During the PCR process, DNA elongation by the polymerase is terminated at the photo-caged nucleotide, and thus this strand becomes by several nucleotide lengths shorter than the counterpart strand as we require. When the protecting group is removed by photoirradiation, predetermined protruding terminal structures are straightforwardly obtained. In the ligation of these PCR products with ARCUT products, no jointing oligonucleotides are necessary as expected. Furthermore, LACE-PCR product and ARCUT scission product are spontaneously (and covalently) connected in the cells due to the long complementary cohesive structure, when both of them are simply incorporated into *E. coli*. Thus, no specific ligase-treatment of the fragments is necessary, making the cloning treatment-free and ligation-independent [18]. Applications of ARCUT to various other types of DNA manipulations have been also evidenced [19,20].

## 6. Homologous Recombination Using ARCUT

**Figure 8:**
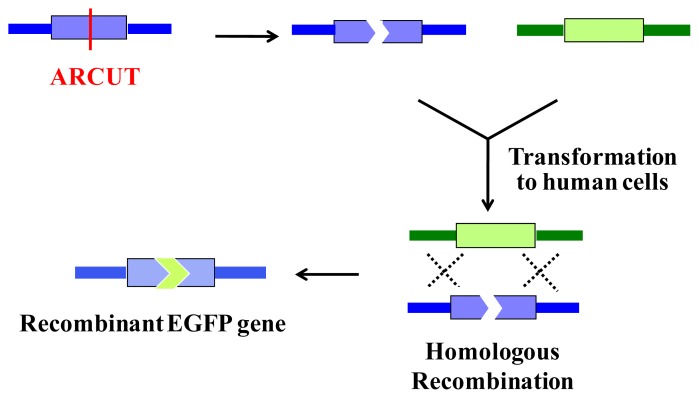
ARCUT-induced homologous recombination for transformation of a gene to desired sequence.

ARCUT is also successfully applicable to homologous recombination in human cells. In this intracellular biological process, a scission site in the genome is repaired by using a donor DNA of homologous sequence as template. Thus, the sequence of the genome around the scission site is varied according to the sequence in the donor DNA used (Figure 8).

**Figure 9:**
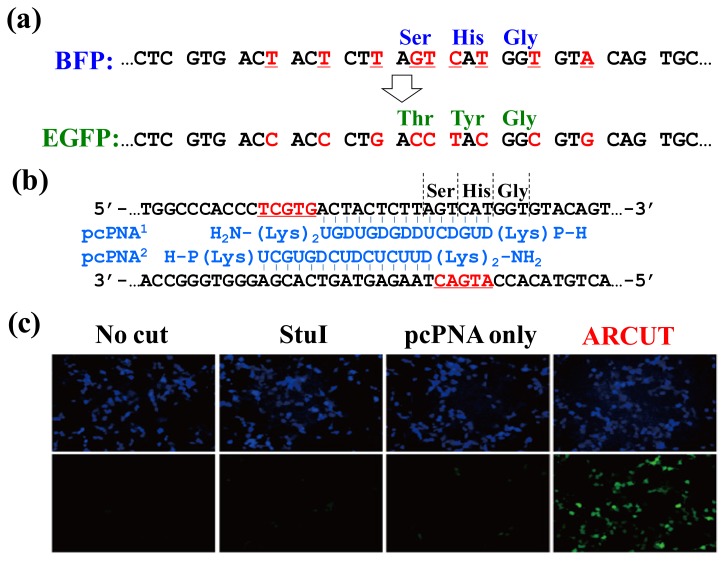
ARCUT-induced homologous recombination in human cells. (**a**) Sequences of *BFP* and *EGFP*. Note that they are almost the same each other except for the chromophore region; (**b**) Structure of the invasion complex; (**c**) Fluorescence microscopy images of the 293T cells. The upper and the lower panels show blue fluorescence emission from BFP and green fluorescence emission from EGFP, respectively.

In Figure 9, the chromophore of blue fluorescent protein (BFP) was converted in human cells to that of EGFP by homologous recombination. The reaction was straightforwardly evaluated in terms of emission of green fluorescence from the cells [21–23]. BFP and EGFP have almost the same primary (and tertiary) structures, and only the amino acids in their chromophores are different (Ser65, His66 and Gly67 for BFP *vs.* Thr65, Tyr66 and Gly67 for EGFP; Figure 9a). The *BFP* gene incorporated in a plasmid vector was cut by ARCUT at the chromophore-coding site (the sequences of *BFP* and the pcPNAs are shown in Figure 9b). In human cells, the product of this ARCUT-scission was introduced together with the donor *EGFP* fragment (742 bp). This donor fragment has no promoter so that it cannot be expressed alone in the cells. Only when the targeted homologous recombination occurs, green fluorescence should be observed. After incubation for two days, many human cells emitted green fluorescence, confirming that the recombination successfully proceeded in the human cells (see the fluorescence microscope images in Figure 9c). Here, the ARCUT scission site in the *BFP* is repaired by DNA polymerase using the *GFP* as a template. Accordingly, the chromophore of the protein produced from the recombinant gene emits the green fluorescence. As expected, without the ARCUT treatment, the recombination was almost negligible. Even when the DNA was cut by a restriction enzyme *Stu*I at a position far from the chromophore-coding site, no homologous recombination occurred. Similarly, the *BFP* gene in adenovirus vector (35 kbp) was successfully converted to *GFP* gene in human cells through the ARCUT-induced homologous recombination. It is conclusive that the site-selective scission by ARCUT is satisfactorily recognized by the repair systems in human cells, even when the scission products have very unique termini structures.

**Figure 10:**
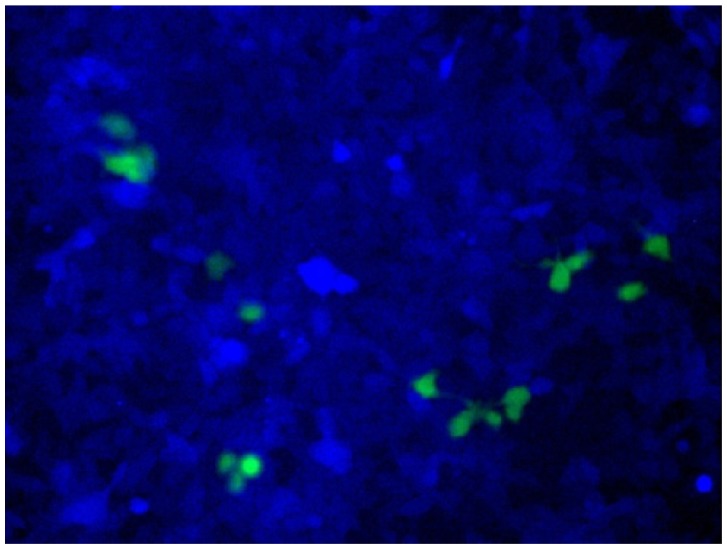
Homologous recombination of *BFP* gene, stably integrated into the human genome, with DNA donor coding a part of *EGFP* gene. To the cells, ARCUT was introduced by electroporation. Note that no cells emitted green fluorescence when only the donor DNA was introduced to the cells.

ARCUT is also applicable to direct engineering of human genome in the cells [24]. As a model system, *BFP* gene was stably integrated to the genome of human cells. In these cells, all the components required for homologous recombination (two pcPNAs, Ce(IV)/EDTA and EGFP donor fragment) were incorporated by electroporation. After several days, some of the human cells emitted green fluorescence, showing that the targeted homologous recombination certainly took place in the human cells (Figure 10). Endogenous genes in human cells can be also converted to desired sequence, simply by designing the appropriate ARCUT. These biological recombinations ultimately provide the same results as “cut-and-paste” method, and, importantly, can be successfully achieved in human cells. Strong potential of ARCUT for future applications has been clearly indicated.

## 7. Conclusions

We recently developed a completely chemistry-based DNA cutter (ARCUT) which selectively cuts double-stranded DNA at designated site with desired specificity. These cutters are composed of Ce(IV)/EDTA complex as molecular scissors and two strands of pseudo-complementary peptide nucleic acid as a sequence-recognizing moiety. The scission-site and site-specificity are freely tuned in terms of the sequences and the lengths of the pseudo-complementary peptide nucleic acid, and thus even human genome can be cut at one predetermined site. Importantly, all the DNA scission proceeds via hydrolysis of the targeted phosphodiester linkages exactly as naturally occurring enzymes cut DNA. Accordingly, the DNA fragments obtained by the site-selective scission of these cutters can be easily ligated by using DNA ligase. This cut-and-paste technology provides a variety of recombinant DNA, which expresses the corresponding protein. The cutter is also applicable to homologous recombination in human cells.

One of the most important advantage of the cut-and-paste methods using ARCUT is that DNA manipulation can be achieved without considering about the presence of appropriate “restriction enzyme sites”. In conventional DNA manipulation, scientists must first find appropriate (naturally occurring) restriction enzymes, which cut the DNA near the manipulation site and then design the whole manipulation project. In many cases, it is difficult to find appropriate “restriction enzyme site” and, in order to choose the second (or the third) choice, some detour strategy is necessary. With the use of ARCUT, however, the choice of scission site and specificity is free. Thus, one can design the shortest pathway to the goal. The tool can be designed simply through the Watson–Crick rule, synthesized by well-established chemical method, and used for the required manipulation. Furthermore, the site-specificity is freely tuned so that even huge genomes can be easily manipulated. The applications of the ARCUT-based cut-and-paste technology should be versatile.

As discussed in the first part of this manuscript, recent developments of protein-based DNA cutters [3,4] are also remarkable. They can be easily introduced to cells by using appropriate vector and prepared *in situ* there, and thus easily employed to various *in vivo* applications. On the other hand, the completely chemistry-based DNA cutters presented here are advantageous with respect to higher flexibility of molecular design, absence of complicated tertiary structure, and possibility of versatile types of functionalization. Apparently, these two types of cutters, which can be the tools to manipulate genomes, have both advantages and disadvantages in the present forms. By choosing appropriate cutter from them according to the needs and taking advantage of the corresponding features of this cutter, the relevant fields should show unprecedentedly remarkable progresses in the near future.
